# Can respondent driven sampling be used to recruit new mothers? A mixed methods study in metropolitan Washington DC

**DOI:** 10.1371/journal.pone.0246373

**Published:** 2021-02-02

**Authors:** Rebecca F. Carlin, Benjamin Cornwell, Jichuan Wang, Yao Cheng, Anita Mathews, Rosalind P. Oden, Linda Y. Fu, Rachel Y. Moon

**Affiliations:** 1 Goldberg Center for Community Pediatric Health, Children’s National Hospital, Washington, District of Columbia, United States of America; 2 Department of Pediatrics, George Washington University School of Medicine and Health Sciences, Washington, District of Columbia, United States of America; 3 Department of Sociology, Cornell University, Ithaca, New York, United States of America; 4 Center for Translational Science, Children’s National Hospital, Washington, District of Columbia, United States of America; 5 Department of Epidemiology and Biostatistics, George Washington University, Washington, District of Columbia, United States of America; 6 Department of Pediatrics, University of Virginia, Charlottesville, Virginia, United States of America; Curtin University, AUSTRALIA

## Abstract

**Background:**

Respondent driven sampling (RDS) is employed to recruit populations that are hard-to-reach, “hidden,” or without a sampling frame. For new mothers (those with infants <6 months) in countries without national health care systems or registries, there is no sampling frame, and random samples may only be attained through costly strategies, e.g., random-dial calling.

**Objective:**

To assess the feasibility of RDS to recruit new mothers.

**Methods:**

In the initial study, we recruited 30 new mothers (“seeds”) from a single birth hospital; each was given 3 referral coupons to give to other mothers (“referrals”). When our sample did not self-perpetuate with referrals, additional seeds were recruited. Demographics of seeds and referrals were compared. A subset of mothers participated in focus groups and were asked about their experience with RDS. We also conducted a second survey of new mothers to further assess feasibility of RDS in this population.

**Results:**

Of the 402 mothers recruited in the initial study, 305 were seeds and only 97 were referrals. Referrals were more likely to be White, highly educated, older, and privately insured (all p≤0.001). Focus group participants indicated that the time required to meet other mothers was an important barrier. In the second survey we recruited 201 mothers; only 53.7% knew ≥1 mother whom they could invite to the study.

**Conclusions:**

New mothers are not easily recruited using RDS because they have a limited number of contacts who are also new mothers. Those recruited through RDS are more likely to be older, Caucasian and of high socioeconomic status, indicating it is not an effective way to recruit a representative sample of new mothers.

## Introduction

Respondent driven sampling (RDS) is an increasingly utilized sampling strategy employed to recruit representative samples of hard-to-reach or “hidden” populations, defined as groups that are a small proportion of the general population and for which there is no comprehensive list of group members (i.e., sampling frame), such that traditional sampling strategies do not work [1, 2]. Hard-to-reach populations that have been successfully recruited via RDS include drug injectors [3], gay and transgender persons [4], and jazz musicians [5, 6]. The approach combines snowball sampling (in which study participants recruit other participants [first “wave”], who then recruit others [second “wave”], etc.; those who successfully recruit other participants receive a small monetary incentive for each participant recruited) [7] with a mathematical model that weights the sample such that the non-randomness of the sampling process is compensated for and the sample is more representative [5]. Recent theoretical and empirical work has assessed the strengths and weaknesses (including the potential for sampling bias) of RDS [8–11].

Mothers of infants <6 months of age (whom we will refer to as “new mothers,” regardless of whether they have older children) are a hard-to-reach or hidden population, particularly in countries without universal health care systems and registries, as there is no sampling frame available for this population. Additionally, in the literal sense, this group is hidden because during the first few weeks of an infant’s life, new mothers are often out of their usual social routines (e.g., if they have maternity leave) and not in public view. From a research point of view, new mothers’ physical contact with the outside world is often limited to clinics and hospitals that are obligated to keep information about them confidential (i.e., hidden); and they are often insulated by their partners and other household members from intrusions by outsiders (e.g., researchers). Thus, truly random samples of this population are difficult to attain in the United States. Random-dial calling is costly because of the low number of households with infants. If the target population is a large proportion of the general population, it can be efficient, but the cost of random-dial calling increases as this proportion decreases [12, 13]. Indeed, the National Opinion Research Center (NORC) at the University of Chicago estimated that, based on their experience with the National Immunization Study, random digit dialing sampling would require 100 household contacts to find one household in our target population, at a cost of approximately $1 million (personal communication, Edward Mulrow PhD, September 24, 2013), making this approach cost-prohibitive. Another possible option is to purchase a list of individuals who, based on advertising data, have new babies. However, it takes several months for individuals’ names and contact information to appear on mailing lists (which are based on infant purchases), and by the time this occurs, it is too late to use these lists to contact these individuals, as their infants have already exceeded the target age of <6 months of age. Because RDS is particularly helpful in hard-to-reach populations that are socially well-connected, we expected that RDS would be an efficient mechanism to recruit new mothers.

In addition to efficiency, RDS provides several additional advantages over other sampling methods. Studies looking at parental practices generally are usually based on convenience samples recruited from selected settings (e.g., hospitals, pediatric primary care sites). Such samples are not representative because the participants are not randomly selected.

RDS uses the bonds of social network members to mobilize networks as a cost-effective means of enrolling subjects [14, 15] and allows for recruitment of a more representative sample of the target population without a sampling frame. The sampling design thus improves representativeness of the sample [16–20]. Furthermore, there is reason to believe that new mothers know and can recruit each other due to their recent potential exposure to each other in parenting classes, play groups, or similar group activities. RDS is superior to conventional chain-referral designs because, by tracking both who recruits whom and how well connected (e.g., by the number of ties) participants are to the target population, RDS estimates can be adjusted for both non-independence among linked respondents and the disproportionate impact of well-connected respondents on sample composition. This is especially true when recruitment chains are allowed to grow beyond a few waves [9]. Finally, some new mothers, particularly U.S. Black mothers, who are mistrustful of medical research are a hidden population from studies, and we hypothesized that RDS may be a means of reaching participants with greater research or medical mistrust. We thus anticipated RDS to be easier and less expensive to implement, and to be more representative of the target population than convenience or simple snowball sampling.

## Methods

This analysis consisted of three parts: 1) a study using RDS methodology to recruit new mothers; 2) focus groups of mothers who had participated in the first study, in which we asked about their experience with RDS; and 3) a separate survey of mothers to assess feasibility of using RDS in this population. The institutional review boards of Children’s National Medical Center and the University of Virginia approved this study; participants provided written informed consent for parts 1 and 2; written consent was waived by the IRBs for part 3.

### Study using RDS recruitment methodology

As part of a larger study recruiting new mothers to investigate the association of social network characteristics and norms with infant sleep practices (details for this larger study are described in [21]), we recruited 30 mothers (“seeds”) from a single birth hospital in Washington, DC, USA to complete a 20–30 minute telephone survey when the infant was at least 2 weeks old, asking about the persons in their personal social networks [22–25], perceived beliefs of network members about infant care practices, and their own infant care practices [26–28]. Each seed received a $20 incentive to participate in the initial survey, with additional monetary incentives if they participated in additional focus groups or surveys. Because the purpose of the original, larger study was to understand whether social network characteristics and norms could help to explain Black-White disparities in infant care practices, inclusion criteria stipulated that all participants self-identify as being Black or White. Mothers also had to be primiparous (first-time) mothers, English-speaking, ≥18 years of age, and living in the metropolitan Washington DC area. As part of the recruitment process, prior to enrollment, each seed was asked if they knew at least 2 “other women like them,” defined as a first-time mother to an infant <6 months old or imminently expecting her first child. Mothers who did not know at least 2 first-time or pregnant women were not enrolled. At enrollment, each seed was then asked to recruit up to 3 other new mothers for the study. She was given 3 “recruitment coupons” coded with numeric digits that confidentially linked her to the person whom she recruited (“referral”) and contained basic information about the study (e.g., purpose of the study and inclusion criteria). Each time a referral enrolled and presented a recruitment coupon, the seed received a $10 incentive, which is the incentive amount provided in original RDS studies [1, 6] and was in addition to the financial incentive received for study participation. While higher incentives were considered, they were considered to be potentially coercive by the institutional review board. Each referral received the standard incentive for enrolling in the study. In addition, the referral, upon study enrollment, also received 3 recruitment coupons linked to her and was offered the same incentives as the seed to stimulate enrollment among network members. When the sample did not self-perpetuate with primiparous referrals, we expanded recruitment to multiparous (not first-time) mothers, increased the maximum number of coupons to 5 upon request for additional coupons, and recruited 30 additional seeds of any parity. When the sample still did not perpetuate, additional seeds of any parity were recruited from the same hospital. All seeds and referrals received coupons to give to other mothers such that RDS and single site recruitment occurred simultaneously. All mothers were recruited between May 2015 and February 2017. Demographics of referrals recruited by RDS were compared to seeds using t-test and Pearson chi-square tests. SPSS v. 23 [29] was used to conduct analyses.

### Focus groups

Qualitative interviews were conducted between July 2016 and January 2018 with a subset of 28 mothers who had participated in the larger study. Focus groups and individual interviews of 1–6 participants/group were stratified by race and parity to increase homogeneity within the individual groups, as studies have shown that homogeneity encourages participants to be honest and forthcoming [30]. Authors met to decide upon interview questions and to develop the interview guide. Interviews were facilitated by trained individuals (RO, AM) and included questions about the RDS process and any challenges related to distributing recruitment coupons. All interviews were video- and audio-recorded and transcribed by a HIPAA-compliant transcription company, after which one author (RO) simultaneously reviewed transcripts and recordings for accuracy. Any disagreement about the transcriptions was resolved through consensus after the other authors independently reviewed the transcripts and recordings. A phenomenological approach was then used to analyze and code the transcripts. Emerging themes and patterns were discussed in regular author meetings, with revisions made iteratively [31]. Findings were verified using concurrent triangulation [32] of the focus group transcripts, the original study findings, and the separate feasibility survey (see below). NVivo 11 plus [33] was used to conduct qualitative analyses.

### Separate survey of mothers to assess feasibility of using RDS

A separate survey was conducted in July through September 2018 of new mothers who presented to a general pediatric clinic or Special Supplemental Nutrition Program for Women, Infants, and Children (WIC) office in Washington DC, USA regarding their contacts to further assess feasibility of RDS among new mothers. Specifically, mothers were asked, “Let’s say that we are recruiting for a study and you are going to be in it and we are looking for other mothers with infants under 6 months of age. How many other mothers do you know who live in this area, and who you would be comfortable inviting to the study?” We asked for the relation of these other mothers (family member, friend, co-worker, etc.) We also collected information about sociodemographics, including age, race/ethnicity, and parity. Frequencies were calculated using Microsoft Excel.

### Patient and public involvement statement

Although participants were not directly involved in study design, recruitment, and conduct, development of the focus group questions was informed by participant responses in the initial study. Further, research questions for the second survey were informed by participant experiences as related in the focus groups. Participants were not invited to contribute to the writing or editing of this manuscript. As all contact information for participants was destroyed after study completion (as per institutional review board approval), study results will not be disseminated to participants.

## Results

### Study using RDS recruitment methodology

**Fig 1:**
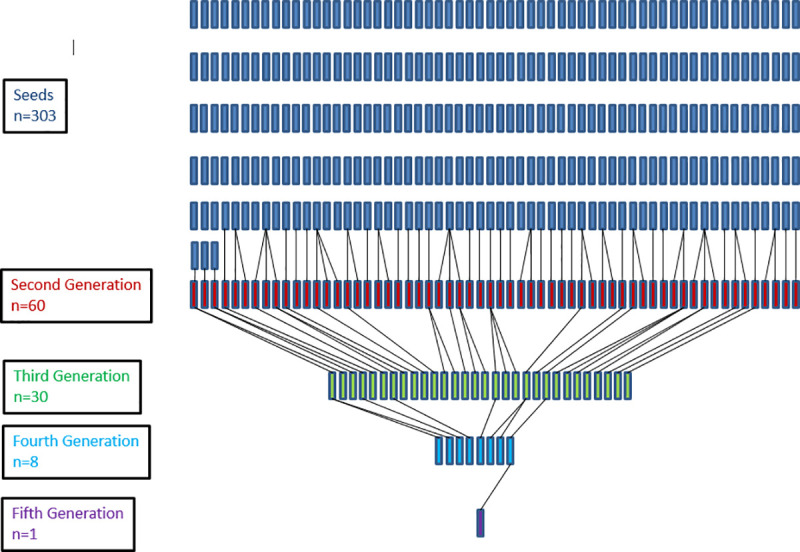
Recruitment networks from RDS sample of mothers in Washington, DC area.

Of the 402 mothers recruited in the initial study, 305 were seeds and 59, 31, 6 and 1 were 2^nd^, 3^rd^, 4^th^ and 5^th^ wave referrals, respectively (Fig 1). When compared to the seeds, referrals were more likely to be White, highly educated, older, and privately insured (all p<0.01) (Table 1).

**Table 1:** Sociodemographic characteristics of the sample by RDS category.

Variable	Seeds (n = 305)	Referrals (n = 97)	P-value*
	N (%)	N (%)	
**Mother’s Age (years), mean (SD)**	28.4 (5.8)	32.0 (5.2)	<0.0001
**Mother’s Race**			<0.0001
Black	229 (75.6)	40 (40.4)	
White	74 (24.4)	59 (59.6)	
**Medical Insurance**			<0.0001
Commercial Insurance and military insurance	158 (52.1)	76 (76.8)	
Medicaid and no insurance	145 (47.9)	23 (23.2)	
Received benefits from the Special Supplemental Nutrition Program for Women, Infants, and Children (WIC)**			0.0014
No	162 (53.5)	71 (71.7)	
Yes	141 (46.5)	28 (28.3)	
**Mother’s Education**			<0.0001
High: Vocational or technical school graduate,4 year college graduate	125 (41.3)	78 (78.8)	
Low: less or equal high school, some college	178 (58.7)	21 (21.2)	

* Seeds and referrals were compared using t-test or Pearson chi-square test.

**Because there is an income requirement (≤185% of U.S. federal poverty level) to receive WIC benefits, receipt of WIC benefits was used as a proxy for low-income status.

### Focus groups

Major themes in the focus groups centered on strategies for coupon distribution and barriers to coupon distribution, including the observation that it takes time for new mothers to meet other mothers of infants and so they might not know mothers who would be eligible. Representative quotes (Q) are in Table 2.

**Table 2:** Qualitative themes and representative quotes (Q).

Themes	Q #	Quote
Successful strategies for distributing coupons	1	*I'm a part of [name of moms’ group]*. *We have a listserv… and I literally just put in the listserv*, *‘Hey*. *Who wants $20 in Target gift cards*?*’ and I got all the responses and*.* *.* *. *I only had five [coupons]*, *so I ran out …It was easy that way*.
	2	*I gave them to my friends and then I had one extra*. *And I put on my listserv…and then I said the first person who responds to me can get the phone number*.
It takes time to meet other new mothers	3	*I think too the other thing that was a challenge for me is that we were right at six months when I did it*. *And so then I think that people who were peers with my son might've timed out too*.
Overlap in mothers’ social networks makes it difficult to find mothers who have not been recruited	4	*I had to work at [finding people to give coupons to] because the person who had given me the coupons kind of gave everybody else the coupons too*, *so there was a slight overlap*.
Mothers did not follow up to see if their referrals enrolled in study	5	*I actually gave mine to friends that I know*, *but I don’t think they [called]*. *I don’t even remember what happened with them*, *sorry*.
	6	*The two or three people that I did give it to*, *I don't even know if they circled back*. *I guess they didn’t because I never got the additional [$10]*, *so I don't know*.
Mothers distributed coupons, but referrals did not enroll in study	7	*For me*, *my people just didn't follow up*. *Three [women whom I gave coupons to]*, *I do know that they needed to be reminded from me*, *like*, *"Hey*. *Did you call*?*" They're like*, *"No*. *Can they call me*?*" I was like*, *"No*.*" For them*, *it was probably—I don't know*, *maybe it's their personality*, *they were the type of people that wouldn't mind getting those hound down calls…*
Coupon distribution not a priority; often forgot to distribute them	8	*I just never…when I was meeting people*, *I wasn't thinking like*, *"Oh*, *they'd be good for the study*.*"*
	9	*I think you sort of know*, *like if your friend is having a baby*. *It's not like I'm not going to keep the coupon for six months down the road to give to somebody*. *So*, *it's either happening or it's not*.

Mothers described giving coupons to and receiving coupons from family members, friends, employees, and teachers. Mothers who were most successful at distributing their coupons used email or listservs to accomplish this (Q1, Q2).

Not knowing other mothers was a common barrier to distributing coupons. Several mothers commented that it would have been easier if their child was older; this would have given them a chance to become acquainted with other mothers through playgroups or similar venues. Mothers also commented that the infant age limit of 6 months was constraining. When they tried to find other mothers, the infants were already too old (Q3).

One mother, who had been recruited by another mother, had difficulty distributing coupons because she did not know any additional mothers who had not already been approached by the first mother (Q4).

Even if the mothers distributed coupons, some acknowledged that they did not follow up to encourage study participation (Q5, Q6). Others did follow up, but their referrals had not contacted study researchers (Q7).

Many mothers commented that they had forgotten to give out coupons. Mothers did not find giving others coupons a priority and often forgot about the coupons after a short period of time (Q8, Q9).

### Separate survey of mothers to assess feasibility of using RDS

In the second survey we recruited a convenience sample of 201 women with infants <6 months of age. Of these, 147 (73.1%) were Black, and 161 (80%) had public (Medicaid) health insurance. Over half (112; 55.7%) were primiparous. Half (108; 53.7%) knew at least one other new mother (median 1 [range, 0–20]) whom they felt comfortable inviting to participate in the study; 15.9%, 17.9%, 7% and 12.9% knew 1, 2, 3, and >3 other new mothers, respectively. Multiparous mothers and primiparous mothers were similarly likely (46% and 60%, respectively) to know other mothers with similarly aged infants (p = 0.052). The vast majority of contacts (69.4%) were friends, followed by family members (23.3%).

## Discussion

Despite our recruiting seeds with pre-identified eligible contacts, RDS was not an effective way to recruit a sample of Black and White, English-speaking, new mothers, regardless of parity. It proved to be difficult for participants to make referrals. Without multi-wave referral chains, sample composition was not converged; thus, sample recruited via RDS overrepresented White women and women of higher socioeconomic status (as indicated by enrollment in private versus public health insurance plans, and non-receipt of WIC benefits). Based on our focus groups and second survey, the reasons for this are likely multifactorial, including a limited number of close contacts with similarly aged infants. Our earlier study found that there is often a shift in women’s social networks after they have their first child such that they gravitate to other women with young children [34], but this study suggests that this shift may take several months, by which time the infants are already >6 months old. Additionally, mothers stated that recruiting referrals for a research study or participating in such a study (e.g., if they received coupons) were not priorities, which is understandable given the unique stress, fatigue and life change that comes with a new infant.

While studies have successfully used RDS to recruit younger adults [35], several researchers have had similar difficulties with perpetuation of chain recruitment [36–38] and resulting biased samples [16, 37]. When recruiting 18–24 year olds online using RDS, Bauermeister et al initially had difficulty perpetuating chains. After interviewing participants about the barriers to referrals, they found that increasing the number of coupons per participant (maximum 5), thus increasing the total possible incentive amounts, and allowing participants to harness online strategies for disseminating coupons (e.g., copying and pasting the link into text messages and social network sites) encouraged participation, and their recruitment rate subsequently increased [37]. Although budget and IRB constraints prohibited increasing incentives per recruit in this study, our participants who asked for additional coupons were given them (maximum 5, thus increasing the total possible incentive amount), and they could photograph coupons to text or email them to contacts rather than transfer them in person to make coupons easier to circulate and less likely to be lost. While this improved perpetuation of chains from the few seeds who were primarily using online groups (e.g., listservs) for referrals, it did not improve the number of referrals from the majority of our participants. Our focus group and interview data indicate that additional steps to encourage prioritization of participation are necessary. The main barriers seemed to be lack of time and the fact that distributing or redeeming referral coupons were not priorities for new mothers, which may be difficult to overcome without substantially increased monetary incentives.

Phillips used RDS to recruit men having sex with men and found that relationship characteristics influenced to whom participants gave their coupons, with people more likely to give coupons to the people with whom they were closest and had the most frequent contact [39]. It is possible that while the women in our study knew other women with similarly aged infants, they may not have had close or frequent contact with these potential referrals and felt uncomfortable recruiting them. By the time close contacts with mothers of similarly aged infants were established (often through mothers’ groups or playdates), the infants were either too old to participate or the coupons were no longer at the forefront of our participants’ minds. Arayasirikul noted that participants were more likely to successfully recruit others if they maintained close ongoing relationships with the study staff [36]. Although our study required 2–3 separate interactions with each seed to administer surveys, at which times they were reminded to distribute their coupons, the nature of our study did not lend itself to close bond formation between study staff and participants, since there were no in-person visits or frequent follow-up visits.

Similar to our findings, others have found that samples recruited through RDS are not always representative of the general population. Both Bauermeister and McCreesh compared the population recruited via RDS to identified representative samples and found that in the RDS samples there was underrepresentation of some groups based on education, race or socioeconomic indicators, and that the selection bias varied, depending on the mode of recruitment (online vs. in person) and the geographic location [16, 37]. Bauermeister et al used web-based RDS recruitment, and their sample over-represented those with higher education and SES [37]. They postulated that, although online recruitment has the advantage of being asynchronous, lower-SES individuals may have limited access to consistent internet access and thus less frequent access to email and social networking applications [37]. Although all seeds in our study had internet access through their cell phones and while they were given paper coupons, it should be noted that several of the women with the highest recruitment numbers used email listservs to identify potential referrals. It is possible that electronic coupons could have been a more effective recruitment strategy. However, it is also possible that women with lower educational and SES levels had less internet access and thus may have been less likely to participate in such groups, and electronic coupons could exacerbate the disproportionately low recruitment of this group. Additionally, it is also possible that seeds would distribute electronic coupons to more than 5 referrals; although each coupon number could only be redeemed once, there is potential for disappointment and frustration among potential referrals and research staff.

We propose two additional reasons that our sample may have been biased towards those who are older and of higher SES. First, women with higher means are likely to have additional supports at home, allowing them the time and resources to meet other new mothers and participate in support groups such as listservs. Second, women of increased financial means may be more likely to participate in a study such as ours for altruistic rather than financial reasons, particularly given our fairly small incentive for referrals. While increasing the financial incentive for recruiting peers may improve the representativeness of the sample, there are also concerns that substantially increased monetary incentives may be perceived as coercive by either participants or ethical review boards.

We acknowledge that this study has several limitations. While our results are consistent with the difficulties others have had using RDS in similar aged populations, our recruitment occurred in a single metropolitan area and only included women who self-identified as Black and White. Thus, this study may not be representative of a larger population. Further, any social networks that included other racial groups may have been interrupted. Also, while the second survey we conducted was recruited from a similar demographic of women in the same geographic region, the samples were not identical, and therefore we can only infer an association between their responses and the difficulty with recruitment in the initial group. Additionally, while we reached thematic saturation in the qualitative portion of the study, qualitative research cannot determine the prevalence of any viewpoint. Therefore, further research is needed to ensure our results are generalizable to the population as a whole. An additional area of research would be a deeper understanding of factors that may make RDS a more successful strategy for recruiting new mothers, and if higher monetary incentives could increase recruitment without seeds feeling coercion. In particular, because we were not able to contact mothers who received coupons but did not enroll in the study, we could not interview them directly to better understand any barriers or facilitators to enrollment.

## Conclusions

Although they are a hard-to-reach population, we found that in the metropolitan Washington DC area, new mothers are not easily recruited using RDS because they often do not know many other new mothers, particularly when recruitment must occur prior to the infant reaching 6 months of age. Even mothers who know other mothers may not prioritize distributing referral coupons or encouraging their referrals to follow through with participation; mothers who receive coupons may not prioritize participating in research studies. Lastly, there is the potential for RDS to create sample biases; those recruited through RDS may be more likely to be older, White and of higher SES than the general population, indicating RDS may not be an effective way to recruit a random sample of new mothers. Traditional recruitment strategies using hospitals or clinics (from which new mothers are not hidden), with specific recruitment goals for specific subpopulations, may be the most feasible approach to recruit a representative, although not random, sample of new mothers.

## Supporting information

S1 File Data for Fig 1. De-identified data regarding waves of recruitment.
(XLSX)

S2 File Data for Table 1. Demographic information about participants.
(XLSX)

S3 File Data for Table 2. Relevant nodes and quotations from the focus groups.
(DOCX)

S4 File Data for the second survey. Data from the separate survey of mothers to assess feasibility of using RDS.
(XLSX)

## References

[1] Heckathorn DD. Respondent-driven sampling: a new approach to the study of hidden populations. Soc Prob. 1997;44:174–99.

[2] Heckathorn DD. Respondent-driven sampling II: deriving valid population estimates from chain-referral samples of hidden populations. Soc Prob. 2002;49(1):11–34.

[3] Heckathorn DD, Semaan S, Broadhead RS, Hughes JJ. Extensions of respondent-driven sampling: a new approach to the study of injection drug users aged 18–25. AIDS Behav. 2002;6(1):55–67.

[4] Ramirez-Valles J, Heckathorn DD, Vazquez R, Diaz RM, Campbell RT. From networks to populations: the development and application of respondent-driven sampling among IDUs and Latino gay men. AIDS Behav. 2005;9(4):387–402. 10.1007/s10461-005-9012-3 . 16235135

[5] Salganik MJ, Heckathorn DD. Sampling and estimation in hidden populations using respondent-driven sampling. Sociol Methodol. 2004;34:193–239. 10.1111/j.0081-1750.2004.00152.x WOS:000229236100009.

[6] Heckathorn DD, Jeffri J. Finding the beat: Using respondent-driven sampling to study jazz musicians. Poetics. 2001;28(4):307–29. 10.1016/S0304-422x(01)80006-1 WOS:000167005400006.

[7] Goodman LA. Snowball Sampling. Ann Math Stat. 1961;32(1):148–70. 10.1214/aoms/1177705148 WOS:A1961WW80800014.

[8] Gile KJ, Beaudry IS, Handcock MS, Ott MQ. Methods for Inference from Respondent-Driven Sampling Data. Annu Rev Stat Appl. 2018;5:65–93. 10.1146/annurev-statistics-031017-100704 WOS:000429191800004.

[9] Gile KJ, Handcock MS. Respondent-driven sampling: An assessment of current methodology. Sociol Methodol. 2010;40:285–327. 10.1111/j.1467-9531.2010.01223.x 22969167 PMC3437336

[10] Goel S, Salganik MJ. Respondent-driven sampling as Markov chain Monte Carlo. Stat Med. 2009;28(17):2202–29. Epub 2009/07/03. 10.1002/sim.3613 . 19572381 PMC3684629

[11] Rocha LEC, Thorson AE, Lambiotte R, Liljeros F. Respondent-driven sampling bias induced by community structure and response rates in social networks. J R Stat Soc a Stat. 2017;180(1):99–118. doi: 10.1111/rssa.12180. WOS:000397117600005

[12] Clagett B, Nathanson KL, Ciosek SL, McDermoth M, Vaughn DJ, Mitra N, et al Comparison of address-based sampling and random-digit dialing methods for recruiting young men as controls in a case-control study of testicular cancer susceptibility. Am J Epidemiol. 2013;178(11):1638–47. 10.1093/aje/kwt164 24008901 PMC3842898

[13] Brogan DJ, Denniston MM, Liff JM, Flagg EW, Coates RJ, Brinton LA. Comparison of telephone sampling and area sampling: response rates and within-household coverage. Am J Epidemiol. 2001;153(11):1119–27. 10.1093/aje/153.11.1119 . 11390332

[14] Heckathorn DD, Broadhead RS, Anthony DL, Weakliem DL. AIDS and social networks: prevention through network mobilization. Sociol Focus. 1999;32:159–79.

[15] Heckathorn DD. Collective sanctions and compliance norms: a formal theory of group-mediated social control. Amer Sociol Rev. 1990;55:366–84.

[16] McCreesh N, Frost SD, Seeley J, Katongole J, Tarsh MN, Ndunguse R, et al Evaluation of respondent-driven sampling. Epidemiology. 2012;23(1):138–47. Epub 2011/12/14. 10.1097/EDE.0b013e31823ac17c 22157309 PMC3277908

[17] Volz E, Heckathorn DD. Probability based estimation theory for respondent driven sampling. J Off Statistics. 2008;24(1):79.

[18] Gile KJ. Improved inference for respondent-driven sampling data with application to HIV prevalence estimation. J Am Statist Assoc. 2011;106:493.

[19] Weinert C. An empirical test of respondent-driven sampling: point estimates, variance, degree measures, and out-of-equilibrium data. Sociol Methodol. 2009;39(1):73–116. 10.1111/j.1467-9531.2009.01216.x 20161130 PMC2743108

[20] Weinert C. Social network analysis with respondent-driven sampling data: a study of racial integration on campus. Soc Networks. 2010;32(2):112–24. 10.1016/j.socnet.2009.09.002 20383316 PMC2850221

[21] Moon RY, Carlin RF, Cornwell B, Mathews A, Oden RP, Cheng YI, et al Implications of Mothers' Social Networks for Risky Infant Sleep Practices. J Pediatr. 2019;212:151–8 e2. 10.1016/j.jpeds.2019.05.027 31201032 PMC6707860

[22] Cornwell B, Schumm LP, Laumann EO, Graber J. Social Networks in the NSHAP Study: rationale, measurement, and preliminary findings. J Gerontol B Psychol Sci Soc Sci. 2009;64 Suppl 1:i47–55. Epub 2009/06/09. gbp042 [pii] 10.1093/geronb/gbp042. 10.1093/geronb/gbp042 19502574 PMC2763519

[23] Marsden P. The reliability of network density and composition measures. Soc Networks. 1993;15(4):399–421.

[24] Marsden P. Egocentric and sociocentric measures of network centrality. Soc Networks. 2002;24(4):407–22.

[25] Marsden P. Survey methods for network data. In: Scott J, Carrington PJ, editors. The Sage Handbook of Social Network Analysis. Thousand Oaks, CA: Sage; 2011 p. 370–88.

[26] Moon RY, Mathews A, Joyner BL, Oden RP, He J, McCarter R Jr. Impact of a Randomized Controlled Trial to Reduce Bedsharing on Breastfeeding Rates and Duration for African-American Infants. J Community Health. 2017;42(4):707–15. 10.1007/s10900-016-0307-2 . 28064421 PMC7327503

[27] Mathews A, Oden R, Joyner B, He J, McCarter R, Moon RY. Differences in African-American Maternal Self-Efficacy Regarding Practices Impacting Risk for Sudden Infant Death. J Community Health. 2016;41(2):244–9. 10.1007/s10900-015-0088-z . 26342946 PMC4779415

[28] Mathews AA, Joyner BL, Oden RP, Alamo I, Moon RY. Comparison of Infant Sleep Practices in African-American and US Hispanic Families: Implications for Sleep-Related Infant Death. J Immigr Minor Health. 2015;17(3):834–42. Epub 2014/04/08. 10.1007/s10903-014-0016-9 24705738 PMC4185304

[29] IBM SPSS Statistics for Windows, Version 23.0. Armonk, NY: IBM Corporation; 2015.

[30] Krueger RA, Casey MA. Focus groups: a practical guide for applied research. 4th ed Thousand Oaks, CA: Sage Publications, Inc.; 2009.

[31] Richards L, Morse JM. Readme first for a user's guide to qualitative methods. 3rd ed Thousand Oaks, CA: Sage Publications, Ltd.; 2013.

[32] Denzin NK, Lincoln YS. Strategies of qualitative inquiry. 4th ed Thousand Oaks, CA: Sage Publications, Ltd.; 2012.

[33] NVivo 11. Melbourne, Australia: QSR International Pty Ltd; 2015.

[34] Moon RY, Mathews A, Oden R, Carlin R. A Qualitative Analysis of How Mothers' Social Networks Are Established and Used to Make Infant Care Decisions. Clin Pediatr (Phila). 2019;58(9):985–92. 10.1177/0009922819845332 . 31018675 PMC8613570

[35] Zembe YZ, Townsend L, Thorson A, Ekstrom AM. Predictors of Inconsistent Condom Use among a Hard to Reach Population of Young Women with Multiple Sexual Partners in Peri-Urban South Africa. Plos One. 2012;7(12). ARTN e5199810.1371/journal.pone.0051998. WOS:000312794500075. 10.1371/journal.pone.0051998 23284847 PMC3527429

[36] Arayasirikul S, Cai X, Wilson EC. A Qualitative Examination of Respondent-Driven Sampling (RDS) Peer Referral Challenges Among Young Transwomen in the San Francisco Bay Area. JMIR Public Health Surveill. 2015;1(2):e9 10.2196/publichealth.4573 27227143 PMC4869213

[37] Bauermeister JA, Zimmerman MA, Johns MM, Glowacki P, Stoddard S, Volz E. Innovative Recruitment Using Online Networks: Lessons Learned From an Online Study of Alcohol and Other Drug Use Utilizing a Web-Based, Respondent-Driven Sampling (webRDS) Strategy. Journal of Studies on Alcohol and Drugs. 2012;73(5):834–8. WOS:000309037600015. 10.15288/jsad.2012.73.834 22846248 PMC3410951

[38] Kuhns LM, Kwon S, Ryan DT, Garofalo R, Phillips G, Mustanski BS. Evaluation of Respondent-Driven Sampling in a Study of Urban Young Men Who Have Sex with Men. J Urban Health. 2015;92(1):151–67. 10.1007/s11524-014-9897-0 25128301 PMC4338125

[39] Phillips G, Kuhns LM, Garofalo R, Mustanski B. Do recruitment patterns of young men who have sex with men (YMSM) recruited through respondent-driven sampling (RDS) violate assumptions? J Epidemiol Commun H. 2014;68(12):1207–12. 10.1136/jech-2014-204206 25086159 PMC4261691

