# Supplementary material for: Can respondent driven sampling be used to recruit new mothers? A mixed methods study in metropolitan Washington DC
Source: PLoS One. 2021 Feb 2;16(2):e0246373. doi: 10.1371/journal.pone.0246373 (PMC7853470; doi:10.1371/journal.pone.0246373)
Supplement: S3 File — Relevant nodes and quotations from the focus groups. (DOCX) [file pone.0246373.s003.docx]

Node list for comments about Coupons:

Feels that she will know more coupon eligible moms later as the network grows with the baby

Gave coupon out to family members

Gave coupon to employee

Gave coupon to friend

Gave coupon to teacher

Gave out coupons but don't know what happened after that

Got coupon from friend

Got coupon through Listserv

Referrals were curious about content of survey

Suggestion for better recruiting, post something on Tacoma Park facebook page

The longer you hang on to the coupon, the less chance of giving them out

Used Listserv to give out coupon

Quotations

Reference 1 - 2.34% Coverage

I have one last question. I know we're running out of time. But with giving out coupons that you all received, was that difficult, easy? Did you find you had to really work at it to get it done or [cross-talking]?

**Victoria:**

I did. I had to work at it because the person who had given me the coupons kind of gave everybody else the coupons too so, there was a slight overlap. But the two or three people that I did give it to, I don't even know if they circled back. I guess they didn’t because I never got the additional, so I don't know. Yeah, that was my experience.

**Moderator:**

If you gave your coupons out and someone called and they qualified for the study, then you would've gotten compensated for each one of those coupons.

**Victoria:**

So, I don't think that it worked. I'm sorry. I gave them out but I don't think --

**Moderator:**

That's okay. That's the luck of the draw. It's okay.

**Dara:**

I'm a part of the Brookland Moms group, we have a LISTSERV -- because I live in Brookland -- and I literally just put in the LISTSERV, "Hey. Who wants $20 in Target gift cards," and I got all the responses and actually ran out of -- I only had five so I ran out and said, "Sorry, guys, I don't have anymore." It was easy that way.

**Yashira:**

For me, my people just didn't follow up. Three, I do know that they needed to be reminded from me, like, "Hey. Did you call?" They're like, "No. Can they call me?" I was like, "No." For them, it was probably -- I don't know, maybe it's their personality, they were the type of people that wouldn't mind getting that hound down call [sounds like], like timeshare [indiscernible].

**Moderator:**

But the thing with this study, we're not allowed to do that with this study. People have to do it voluntarily.

**Victoria:**

I think too the other thing that was a challenge for me is that we were right at six months when I did it and so then I think that people who were peers with my son might've timed out too. I think that might've been a part of it as well.

**Staff:**

All right. That's it for me.

[<Internals\\SONIC FG 022517 white2+>](file:///C:\Users\rym4z\AppData\Local\Microsoft\Windows\Temporary%20Internet%20Files\Content.Outlook\ZRSV28KX\2171221c-337f-466c-bbd4-d853cd71b6da) - § 2 references coded [0.96% Coverage]

Reference 1 - 0.56% Coverage

And the person that you received the coupon from, is that person somebody that you really trust or is it just somebody [cross-talking]?

**Catherine:**

I don’t actually know her.

**Mary:**

Wait, what?

**Catherine:**

It was through LISTSERV, the coupons.

**Mary:**

Oh, yes, from the LISTSERV. Yeah, that’s right. So I have no idea who it was.

Reference 2 - 0.40% Coverage

And you had no trouble giving out your coupons --?

**Catherine:**

I actually gave mine to friends that I know, but I don’t think they were given --

**Mary:**

I don’t even remember what happened with them, sorry.

**Facilitator:**

It’s okay.

[<Internals\\SONIC FG 032517 aa2+>](file:///C:\Users\rym4z\AppData\Local\Microsoft\Windows\Temporary%20Internet%20Files\Content.Outlook\ZRSV28KX\3e37d445-b792-402f-80d4-d853ccfaa3f4) - § 1 reference coded [0.13% Coverage]

Reference 1 - 0.13% Coverage

Or did you get a coupon from someone else?

**Ayesha:**

I had a coupon from one of my girlfriends, and that’s why.

[<Internals\\SONIC FG 052017 aa1>](file:///C:\Users\rym4z\AppData\Local\Microsoft\Windows\Temporary%20Internet%20Files\Content.Outlook\ZRSV28KX\afa8bae4-3206-4d16-8cd4-d853cc62396e) - § 1 reference coded [0.24% Coverage]

Reference 1 - 0.24% Coverage

That’s why we gave the coupon, so that we could give coupons out to people like yourselves to make --

**Jessica:**

Yeah. I gave them to a few, to other moms. I don’t know if they gave --

**Moderator:**

But you never can tell how people are really going to respond.

**London:**

Yeah.

[<Internals\\SONIC FG 070916 white1>](file:///C:\Users\rym4z\AppData\Local\Microsoft\Windows\Temporary%20Internet%20Files\Content.Outlook\ZRSV28KX\9a00848e-cbc9-471d-9ad4-d853cbbb8566) - § 2 references coded [1.65% Coverage]

Reference 1 - 0.28% Coverage

Yeah. I think you sort of know, like if your friend is having a baby. It's not like I'm not going to keep the coupon for six months down the road to give to somebody. So, it's either happening or it's not.

Reference 2 - 1.38% Coverage

Do you feel like when, like once you had your child and you started to make a little bit more of a network or talk to people more who had younger children, you had more possibilities to give the coupons to than you would have when you were in the hospital?

**Shanti:**

Yeah. Because I think one of the people I thought of -- I don't remember if we had to give names or just had to think of who they were in the hospital but one of the --

**Rebecca:**

I would take names just to,

**Shanti:**

Because one of the persons I thought of I think was born in England. I mean, I know she was born in England. I think she was one of the persons I thought of in the hospital but then realized later she wouldn't be eligible because -- anyway, so I wasn't -- it was definitely better to think about it later.

**Rebecca:**

You had more possibilities later on?

**Shanti:**

Yeah, for sure,

**Andrea:**

I guess I had two. I just never -- I don't think when I was meeting people; I wasn't thinking like, "Oh, they'd be good for the study."

[<Internals\\SONIC FG 112616 aa2+>](file:///C:\Users\rym4z\AppData\Local\Microsoft\Windows\Temporary%20Internet%20Files\Content.Outlook\ZRSV28KX\d9c26ee4-8b1c-42f6-aed4-d853cab32c1f) - § 7 references coded [1.42% Coverage]

References 1-2 - 0.32% Coverage

For those of you that gave out your coupons to other people, were they friends or were they just acquaintances?

**Nichita:**

No, it was my friend.

**Latisha:**

I gave mine to my sister.

**Karen:**

For me, it was my two sisters-in-law.

References 3-5 - 0.09% Coverage

I gave it to one of my employees, one of my teachers, and my friend.

Reference 6 - 0.18% Coverage

I’m just curious to know whether those referrals asked you about what’s being asked in the survey.

**Karen:**

One of them asked me.

Reference 7 - 0.84% Coverage

Did you brief them about what the survey is about, what topics, what kinds of questions are being asked? Is that kind of giving them an advantage about how to answer these questions and what to expect?

**Karen:**

No. Well, when you give someone a coupon, you definitely want to tell them what is it for. It wasn’t going through each question. I gave the coupon. One of them said, oh, okay. She sits there and said, oh, okay. The other one said, well, what is it for? I just said, oh, they’re just going to ask you some questions because they want to prevent SIDS. That was just basically it. I didn’t go through each question with them.

[<Internals\\SONIC II 072316 white2+>](file:///C:\Users\rym4z\AppData\Local\Microsoft\Windows\Temporary%20Internet%20Files\Content.Outlook\ZRSV28KX\64ceb211-de4c-454d-b2d4-d853c97b33b1) - § 3 references coded [3.79% Coverage]

Reference 1 - 0.61% Coverage

**Facilitator**:

Yeah. So, was it easy for you to just give out your coupons when you got them in the mail?

**Susan:**

Yes, I did. I gave them to my friends. Because I think I only had a couple or three.

**Facilitator**:

It should've been three.

Reference 2 - 0.74% Coverage

Oh, yeah. Okay. This is what happened. I gave them to my friends and then I had one extra and I put on my LISTSERV, explained what I was told about what the demographic had to be and then I said the first person who responds to me can get the phone number. I think that's how I handled it.

Reference 3 - 2.45% Coverage

So, yes, we've been brainstorming about how we can be more active and get more people involved but we have to kind of rely on the rules of the study. I've even thought about like Google, we can do a Google chat but then that's HIPAA. Suppose you don't want your face seen;

**Susan:**

Well, if you put something on the Tacoma Park moms' Facebook page with an invitation, is that;

**Facilitator**:

No, that will be good. Because then people could contact us. Yeah. I'm sure Brandy is working on that.

**Susan:**

Yeah. And if you need, since I'm on the Facebook page and I'm a member, I'm happy to post anything that you want to do.

**Facilitator**:

Okay. So maybe I'll send you the flyer, the original flyer.

**Susan:**

I can't add a, well yeah, I could do that.

**Facilitator**:

Or just the information; Once you see the flyer, you can just say what it is.

**Susan:**

Cut and paste it. Yeah. Okay. I'll do it.

**Facilitator**:

Okay awesome.

**Susan:**

I'd be happy to do that.
